# Durable antiviral protection via direct *in vivo* programming of hematopoietic stem cells

**DOI:** 10.1016/j.omta.2026.201814

**Published:** 2026-07-24

**Authors:** Anja Ehrhardt

**Affiliations:** 1 Virology and Microbiology, Center for Biomedical Education and Research (ZBAF), School of Medicine, Faculty of Health, Witten/Herdecke University, Witten, Germany

## Main text

Efforts to achieve durable control or eradication of HIV-1 have increasingly turned toward gene-based strategies potentially capable of providing long-term protection. In this context, a new study by Li et al.^1^ presents a notable conceptual advancement: the direct *in vivo* genetic programming of hematopoietic stem cells (HSCs) to generate a systemic source of antiviral biologics. By exploring helper-dependent adenoviral vectors (HDAd), the authors bypass the logistical and biological constraints associated with *ex vivo* HSC manipulation.

Direct *in vivo* engineering of HSCs gained increasing interest for gene therapeutic approaches. The first approach was introduced in 2016 using an adenoviral vector.^2^ Later, in 2023, another study explored lipid nanoparticles as delivery vehicle,^3^ and lentiviral vectors and adeno-associated viral (AAV) vectors were investigated for *in vivo* HSC transduction. The first study performed in 2016 was based on a capsid-modified HDAd for *in vivo* HSC gene transfer. Here, the authors used the HDAd 5/35++ vector platform based on human adenovirus (Ad) type 5 (Ad5) carrying an optimized fiber (F35++) derived from human Ad type 35 (Ad35) for improved CD46 binding. This vector platform for stable transduction of target cells based on somatic integration mediated by the *Sleeping Beauty* transposase integration machinery was explored for stabilized *in vivo* stem cell transfer and treatment of murine thalassemia intermedia.^4^ Afterward, the system was further developed for precise genome engineering.^5^ In the new study by Li et al., the authors used a novel HDAd 6/3+ platform based on Ad type 6 (Ad6) carrying an optimized Ad type 3 (Ad3) fiber (F3+), which was also explored before using the fiber F35++.^6^ The switch from the HDAd 5/35++ platform to the HDAd 6/3+ platform is advantageous, as the Ad5-based vectors are restricted by pre-existing immunity. In contrast to Ad5, Ad6 is a rather rare virus with significantly lower seroprevalence in the human population. Besides changing the vector backbone, the authors also exchanged the fiber knob from Ad35 (F35++) for CD46 binding to Ad3 (F3+) for desmoglein-2 (DSG-2) targeting.^7^ Using this approach, unwanted vector binding to erythrocytes in non-human primates (NHPs) can be avoided. Moreover, DSG-2 binding results in specific transduction of both NHP and human cells. Milestones for the HDAd vector platform development are summarized in Table 1.

**Table 1:** Milestones of the HDAd vector development for direct *in vivo* generation of modified hematopoietic stem cells to combat HIV

Milestone	Approach and major result	Vector type	Year/Reference
First *in vivo* HSC transfer using HDAd	a CD46 targeting vector was used to stably transduce mobilized HSCs	HD-Ad5/35^++^ SB transposase for somatic integration	2016/Richter et al.^2^
First correction of a genetic disease after direct *in vivo* transduction of HSC	a CD46 targeting vector based on HD-Ad5/35^++^ corrects murine thalassemia intermedia	HD-Ad5/35^++^ SB transposase for somatic integration	2018/Wang et al.,^4^
First description of the HD-Ad5/3+ vector platform in NHPs	a DSG2 targeting vector that is not sequestered by erythrocytes in NHPs	HD-Ad5/3^+^ SB transposase for somatic integration	2022/Wang et al.^7^
*In vivo* HSC transfer using the HDAd6 vector platform	a CD46 targeting vector based on Ad6 with lower seroprevalence was explored	HD-Ad6/35^++^ SB transposase for somatic integration	2023/Wang et al.^6^
Expression of eCD4-Ig using the HD-Ad5/35^++^ platform	first study using a HDAd5/35++ vector expressing rhesus eCD4-Ig for *in vivo* HSC engineering in macaques. No SIV elimination was observed.	HDAd5/35++ SB transposase for somatic integration	2023/Li et al.^9^
Expression of eCD4-Ig using the HD-Ad6/3^+^ platform in NHP	first proof-of-concept study for SIV prevention in NHPs based on the Ad6/3^+^ platform for expression of an improved eCD4-Ig variant	HDAd6/3+ SB transposase for somatic integration	2026/Li et al.^1^

Abbreviations: HSC, hematopoietic stem cell; HDAd, helper-dependent adenoviral vector; Ad, adenovirus; DSG2, desmoglein; NHP, non-human primate; SB transposase, *Sleeping Beauty* transposase; SIV, simian immunodeficiency virus.

Central to this work is the sustained expression of eCD4-Ig-Emm06, an engineered decoy that recapitulates key features of the CD4 receptor and coreceptor interactions required for viral entry. The parental decoy protein eCD4-Ig was first explored in 2015 using AAV-vector-mediated muscular delivery in NHPs^8^ and in 2023 for direct *in vivo* HSC transduction using the HDAd5/35++ vector platform.^9^ eCD4-Ig is an Fc-fusion protein comprising CD4 domains 1 and 2 fused to an immunoglobulin G (IgG) Fc, with a sulfated CCR5-mimetic peptide at the C-terminus. It inhibits infection by binding the CD4 and coreceptor binding sites on the HIV-1 or simian immunodeficiency virus (SIV) envelope glycoproteins. However, the latter vector approach using eCD4-Ig was unsuccessful in eliminating SIV in NHPs, at least in part due to development of antibodies against the transgene product. In the new study, the rhesus eCD4-Ig variant (“eCD4Ig-Emm06”) was used, containing half-life extending substitutions in the rhesus IgG2 Fc and substitutions in rhesus macaque CD4 that enhance stability, neutralization potency, and *in vivo* half-life. Additionally, rhesus tyrosylprotein sulfotransferase 2 (TPST2) was co-expressed to ensure sulfation of the CCR5-mimetic peptide, and CXCR4 was co-expressed to enhance homing of mobilized HSC to bone marrow.

To deliver eCD4-Ig-Emm06 using HDAd 6/3+ as delivery platform, the authors initially demonstrate stabilized expression of eCD4Ig-Emm06 in mice also after transfer of modified HSCs into lethally irradiated secondary mice. Next, the authors explored efficacy of the system in a non-human primate (NHP) model and show that cytokine prophylaxis effectively suppressed inflammatory responses directly after vector infusion. However, in contrast to mouse experiments, only two instead of four *in vivo* selection cycles of transduced cells using O6-benzylguanine/carmustine (O6BG/BCNU) could be performed in NHPs because of BCNU-associated pneumonitis. Therefore, the current *in vivo* selection strategy remains a significant barrier to clinical translation, necessitating the development of safer alternatives before broader implementation of this approach can be considered.

Next, the authors demonstrate that *in vivo*-generated eCD4Ig-Emm06 was highly functional in neutralization efficacy of HIV- and SIV-derived envelope glycoproteins after NHP-vector infusion and subsequent selection. Interestingly, they also show that immunosuppression can suppress immune responses against the transgene in absence of SIV infection. Biodistribution after *in vivo* modification of HSCs in NHPs revealed that the spleen was the preferred homing organ for modified HSPs after two selection cycles. Furthermore, on the vector genome copy-number level, CD3+ T cells and CD20+ B cells were the preferred cells in peripheral blood mononuclear cells (PBMCs) and bone marrow, in which the vector genome was detectable. Interestingly, secretion of the transgene was measurable in two treated animals in B cells and in one animal from monocytes, but not in T cells. Collectively, these results indicate that HDAd-transduced HSCs generated both lymphoid and myeloid lineages that subsequently migrated to systemic tissues. These results are a particularly compelling aspect because it can be concluded that transduced HSCs retain multilineage differentiation capacity, and the identification of B cells as a major contributor to circulating eCD4-Ig-Emm06 adds an intriguing layer of biological synergy.

To evaluate the protective potency of expressed eCD4Ig-Emm06 in NHPs after *in vivo* HSC gene transfer, SIV challenge experiments were performed, suggesting that viral replication can be suppressed and that disease progression of the infectious disease can be slowed down. Note that higher eCD4Ig-Emm06 expression levels were advantageous. Importantly, 7 and 11 weeks post-infection with SIV, there was no evidence of virus escape mutants. After SIV challenge, results obtained from treated animals suggested reduced viral loads in the spleen, which correlated with the highest concentration of transduced cells in this organ. Moreover, high levels of eCD4Ig-Emm06 seems to result in a reduced number of bar-coded SIV variants establishing infection, a delayed onset of viremia, lower viral loads in plasma, and preservation of CD4^+^CCR5^+^ T cells. In total, the demonstration of long-term expression in rhesus macaques, alongside preserved neutralization potency *in vivo*, reinforces the therapeutic potential of this molecule when delivered via a durable cellular source.

**Figure 1:**
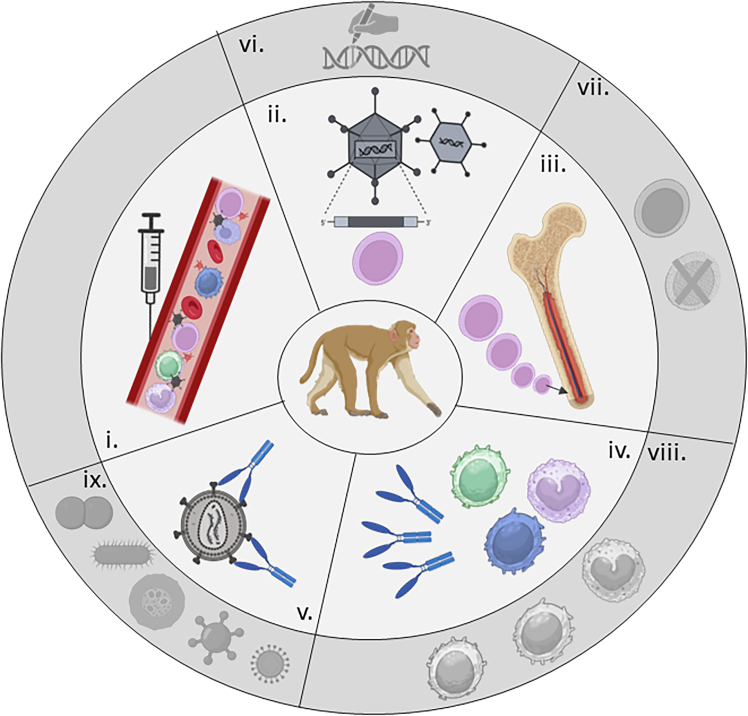
Overview of involved steps for direct *in vivo* hematopoietic stem cell (HSC) transduction for stable expression of secreted biotherapeutics (i) The vector is applied systemically after mobilization of HSCs into peripheral blood. (ii) HSCs are transduced, and the transgene stably integrates into the host genome. (iii) Modified HSCs reside in the bone marrow, and modified cells are selected. (v) Modified cells secrete the biotherapeutic molecule. (vi) The secreted molecule neutralizes SIV. The strategy can be improved and extended with respect to (vi) genome editing tools for targeted genome engineering of HSCs, (vii) selection optimization of transduced cells, (viii) lineage-specific expression of biotherapeutics, and (ix) broad protection against other microorganisms.

Overall, Li et al. demonstrate that the HDAd 6/3+ platform is a promising *in vivo* gene therapy approach to deliver biologic therapeutics. The complete pipeline is schematically shown in Figure 1. Based on this work, future studies can utilize this knowledge to implement lineage specific expression, minimize vector and transgene immunogenicity, and optimize selection of genetically modified cells. Further potential of applications and improvements of the approach are also displayed in Figure 1. Especially investigations related to lineage-specific expression of secreted biotherapeutics such as antibodies in general or bispecific molecules may direct the field toward novel therapeutic strategies. In the current study, the *Sleeping Beauty* transposase system was explored for somatic integration of the therapeutic and protective transgene into HSCs. However, in the future, a combination with efficient site-directed genome engineering tools, including CRISPR/Cas based systems, may be preferentially used to avoid potential genotoxicity.

The study provides a proof of concept for *in vivo* HSC gene therapy as a platform for sustained delivery of antiviral biologics. Most importantly, this approach can be expanded to broadly apply it to other complex infectious diseases. However, important hurdles remain. Larger cohorts of treated large animals (>3) need to confirm observations, other *in vivo* selection strategies with less side effects should be explored, and potentially better outcomes are expected if bone marrow homing of modified HSCs could be enhanced. Note that a previous study used intramuscular administration of AAV as alternative delivery platform for eCD4-Ig,^8^ also resulting in long-term expression of the neutralizing biotherapeutic. Thus, potentially direct comparisons utilizing different administration routes resulting in different target cells for long-term expression of antiviral molecules need to be evaluated. Regardless of which vector system and administration route are applied to deliver neutralizing agents, mucosal challenge of the infectious agents also remains to be investigated. While further optimization and investigations are needed, the integration of vector engineering, stem cell biology, and antiviral design presented in this study represents a step toward long-acting therapies for HIV and other chronic infectious diseases using a single-shot functional cure.

## Acknowledgments

The figure has been created with BioRender.com.

## Declaration of interests

The author declares no financial conflicts of interest. However, the commentary discusses the HDAd platform and cites previously published studies on which the author was a co-author.
